# Trends in the Prevalence of Non-Alcoholic Fatty Liver Disease and Its Future Predictions in Korean Men, 1998–2035

**DOI:** 10.3390/jcm9082626

**Published:** 2020-08-13

**Authors:** Seo Young Kang, Ye-Jee Kim, Hye Soon Park

**Affiliations:** 1 International Healthcare Center, Asan Medical Center, Seoul 05505, Korea; sykang@amc.seoul.kr; 2 Department of Clinical Epidemiology and Biostatistics, Asan Medical Center, Seoul 05505, Korea; kimyejee@amc.seoul.kr; 3 Department of Family Medicine, Asan Medical Center, University of Ulsan College of Medicine, 88, Olympic-ro-43-gil, Songpa-gu, Seoul 05505, Korea

**Keywords:** non-alcoholic fatty liver disease, obesity, abdominal obesity, unhealthy lifestyles

## Abstract

Non-alcoholic fatty liver disease (NAFLD) is a serious health concern as it can progress to liver cirrhosis and hepatoma. We investigated past trends in the prevalence of NAFLD and related factors among Korean men and women from 1998 to 2017 and predicted their future prevalence among Korean men. We used data from the Korea National Health and Nutrition Examination Survey I–VII (KNHANES). NAFLD was defined as a hepatic steatosis index of >36. Subjects with viral hepatitis, liver cirrhosis, cancer, pregnancy, and a habit of drinking ≥30 g alcohol per occasion were excluded. We evaluated the prevalence trends of NAFLD, obesity, abdominal obesity, high fat intake, and low physical activity in each KNHANES wave. For future prevalence predictions, average annual percentage changes (AAPCs) were estimated from the joinpoint model. In men, NAFLD prevalence has increased by approximately 11 percentage points in the past 19 years, reaching 30.7% in wave VII. Prevalence of obesity, abdominal obesity, high fat intake, and low physical activity also increased. The AAPC of NAFLD prevalence was 2.3% per year, and the estimated NAFLD prevalence in 2030 and 2035 was 39.1% and 43.8%, respectively. The forecasted prevalence of obesity, abdominal obesity, and high fat intake among Korean men in 2035 was 65.0%, 52.2%, and 23.5%, respectively. The estimated future prevalence of NAFLD and related factors was considerably high in the younger age group (19–45 year). In women, NAFLD prevalence has increased by approximately three percentage points in the past 19 years; however, this increase was not significant in the multivariate analysis. Public strategies to manage obesity, abdominal obesity, and unhealthy lifestyles are needed to prevent NAFLD.

## 1. Introduction

Non-alcoholic fatty liver disease (NAFLD) is a serious health concern as it may progress to liver cirrhosis and hepatocellular carcinoma (HCC). The spectrum of NAFLD varies according to individual risk factors and clinical conditions; however, annually, 0.25–3.2% of the population develop cirrhosis from NAFLD and 0.3–2.6% develop HCC from cirrhosis [1]. The prevalence of NAFLD in Asia was estimated to be 27%, which was not lower than the global prevalence of 25% [2]. The prevalence of NAFLD in Korea was reported to be approximately 20 to 50% depending on the diagnostic modality and study population [3,4,5]. One of the clinical implications of NAFLD is that persons with this condition may eventually require liver transplantation after developing cirrhosis and HCC [6]. NAFLD-related liver disease is the most rapidly increasing indication for liver transplantation in the US, and patients with this disease are more susceptible to cardiovascular complications and sepsis after transplantation than those without [7,8].

There are sex differences in the prevalence, risk factors, and clinical outcomes of NAFLD. In general, the prevalence of NAFLD is higher in men than in women, and men have worse clinical outcomes [9]. A multi-national cohort study conducted among patients with biopsy-confirmed NAFLD showed that the overall mortality, transplantation rate, and incidence of HCC were higher in men [10]. The risk for HCC was about twofold higher in men, and men developed HCC at earlier fibrosis stages than women [10,11].

Obesity, abdominal obesity, unhealthy dietary factors, and low physical activity are well-recognized risk factors for NAFLD. Worldwide, the prevalence of obesity and the prevalence of NAFLD are highly correlated with each other, and abdominal obesity increases the NAFLD risk even after adjustments for body mass index (BMI) [12,13]. Excessive fat intake plays a key role in NAFLD development, and a sedentary lifestyle is associated with NAFLD [14]. The increasing prevalence of obesity and abdominal obesity along with a Westernized diet and low physical activity contributes to the increasing prevalence of NAFLD in Asia.

Such an increase in the prevalence of NAFLD has serious implications for public health, and formulation of comprehensive public strategies will be required to prevent NAFLD. To cope with the increasing prevalence of NAFLD, it is important to understand the past and future trends of NAFLD and its risk factors. Therefore, in this study, we investigated the trends in the prevalence of NAFLD and associated risk factors among Korean men and women using a nationally representative sample. More specifically, we evaluated the trends in the prevalence of NAFLD, obesity, abdominal obesity, high fat intake, and low physical activity from 1998 to 2017. Furthermore, we predicted the future prevalence of NAFLD and associated factors up to 2035 among Korean men.

## 2. Materials and Methods

### 2.1. Data Source and Study Population

We used the data from the Korea National Health and Nutrition Examination Survey (KNHANES) wave I–VII (1998–2017). The KNHANES, conducted by the Korea Centers for Disease Control and Prevention, is a nationwide representative cross-sectional survey that applies stratified, clustered, multistage probability sampling based on geographic area, age, and, sex. The KNHANES consists of seven waves as follows: wave I (1998), wave II (2001), wave III (2005), wave IV (2007–2009), wave V (2010–2012), wave VI (2013–2015), and wave VII (2016–2018). The sampling strategies and survey items are slightly different in each wave. Additional information about the study design and methodology has been previously reported [15]. Of the 200,713 participants in KNHANES I–VII, we excluded those with age <19 years (*n* = 51,129), with hepatitis and/or liver cirrhosis (*n* = 4272), with cancer (*n* = 3211), with a habit of drinking ≥30 g alcohol per occasion (*n* = 37,659), with pregnancy (*n* = 532) or with missing responses precluding the determination of alcohol status (*n* = 94,744), and with missing values precluding the evaluation of NAFLD status (*n* = 119,686). With regard to hepatitis and/or liver cirrhosis status, participants were asked to report whether they had been diagnosed with hepatitis B, hepatitis C, and/or liver cirrhosis from a physician. Concerning alcohol consumption, participants were asked to provide the amount of alcohol drinking per occasion in the past year. After the exclusions, a total of 10,870 men and 30,078 women were included in the final analyses. All participants provided written informed consent before participating in the KNHANES. The study protocol conforms to the ethical guidelines of the 1975 Declaration of Helsinki as reflected in a priori approval by the institution’s human research committee.

### 2.2. Assessment of Lifestyle Characteristics

Dietary variables were evaluated using the single-day 24-h recall method. Trained dieticians visited each participant’s residence and conducted in-person interviews using a food consumption table. On the basis of the interview responses, the total energy intake (kcal/day) and the daily carbohydrate, protein, and fat intake of each participant were calculated. High fat intake was defined as a fat intake of ≥30% of the total energy intake. As a measure of physical activity, the International Physical Activity Questionnaire (IPAQ) was used for the 2005–2013 data, and the Global Physical Activity Questionnaire (GPAQ) was used for the 2014–2017 data. Low physical activity was defined as inactive status in IPAQ and low physical activity in GPAQ [16,17]. With respect to alcohol use, we categorized the study participants into those who drink alcohol <30 g per occasion and non-drinkers. Smoking status was categorized as current smoking and non-smoking. Household income was divided into quartiles, and the lowest quartile was defined as low income in the questionnaire. Educational level was categorized as <12 and ≥12 years.

### 2.3. Measurement of Metabolic Variables

All anthropometric and laboratory variables were obtained using standardized equipment and techniques. Height was measured to the nearest 0.1 cm in the standing position with a portable stadiometer, and weight was measured to the nearest 0.1 kg using a balanced scale. BMI was calculated by dividing body weight by the square of height (kg/m^2^). Obesity was defined as a BMI of ≥25 kg/m^2^ [18]. Waist circumference was measured to the nearest 0.1 cm at the midpoint between the top of the iliac crest and the lowest rib. Abdominal obesity was defined as a waist circumference of ≥90 cm [19]. Systolic blood pressure and diastolic blood pressure were measured three times with 5-min intervals with a sphygmomanometer. We used the average of the second and third measurements in the analysis. Blood samples were collected after fasting for at least 8 h, and laboratory values were evaluated in a certified laboratory.

### 2.4. Definition of NAFLD

NAFLD was defined according to the hepatic steatosis index (HSI), which is a simple screening tool for diagnosing NAFLD [20]. The HSI has sensitivity and specificity for detecting NAFLD of 93.1% and 92.4%, respectively, and has been used in previous studies in different countries [20,21,22]. NAFLD was defined as present when the HSI (8 × (alanine aminotransferase/aspartate aminotransferase ratio) + BMI (+2 if female, +2 if diabetes mellitus)) was >36. With regard to diabetes mellitus, participants with fasting glucose ≥126 mg/dL, those who were diagnosed with diabetes mellitus by a physician, or those who were taking oral hypoglycemic agents or receiving insulin treatment were considered to have diabetes mellitus.

### 2.5. Statistical Analysis

All analyses were performed after accounting for the complex sample design and sample weights to represent the Korean population. Descriptive statistics were used to present the study variables in each wave of KNHANES. Means and standard errors (SEs) are presented for continuous variables, and unweighted numbers and weighted percentages are presented for categorical variables. To compare the basic characteristics between the present study population and all the participants in KNHANES, we presented the prevalence for the main variables in each wave of KNHANES among two different populations. The trends in the prevalence of NAFLD and associated factors in each wave of KNHANES are presented as unweighted numbers and weighted percentages. The odds ratios (ORs) and 95% confidence intervals (CIs) for NAFLD and associated factors in each wave of KNHANES were calculated using multivariate logistic regression analyses. We presented the *p*-values for trend according to the wave of KNHANES in each analysis. The analyses were performed separately for men and women.

To forecast the future prevalence of NAFLD and each associated factor among men, we calculated the age-adjusted prevalence (%) of NAFLD and each associated factor considering the population age distribution shift. To measure the trend transition during the 19-year period, the average annual percentage changes (AAPCs) were estimated as the weighted averages of the annual percentage changes from the joinpoint model using Joinpoint Regression Program version 4.6.0.0 (Statistical Methodology and Applications Branch, Surveillance Research Program, National Cancer Institute, Rockville, MD, USA) [23,24]. All analyses were conducted with IBM SPSS Statistics for Windows version 23.0 (IBM Corp., Armonk, NY, USA) and SAS software version 9.3 (SAS Institute, Cary, NC, USA).

## 3. Results

### 3.1. Basic Characteristics of the Study Participants

Table 1 shows the basic characteristics of the 10,870 men and 30,078 women. In men, height has increased by about 0.7 cm, and weight has increased by about 0.6 kg over the past 19 years. BMI has increased by about 2 kg/m^2^, and waist circumference has increased by about 5 cm. The total energy intake has not changed; however, the proportion of fat intake has increased. The proportion of participants with low physical activity has more than doubled, and the proportions of current drinkers and smokers have decreased. In women, height has increased by about 0.4 cm, and weight has increased by about 0.3 kg over the past 19 years. BMI has increased by about 0.4 kg/m^2^, and waist circumference has increased by about 1.3 cm. The amount of total energy intake and carbohydrate intake have decreased; however, the proportion of fat intake has increased. The proportion of participants with low physical activity has more than doubled, and the proportion of current smokers has decreased. Table 2 shows that the prevalence and trends of the basic characteristics are comparable between the present study population and the entire study participants in KNHANES.

### 3.2. Trends in the Prevalence of NAFLD and Associated Factors in Korean Men and Women

**Figure 1:**
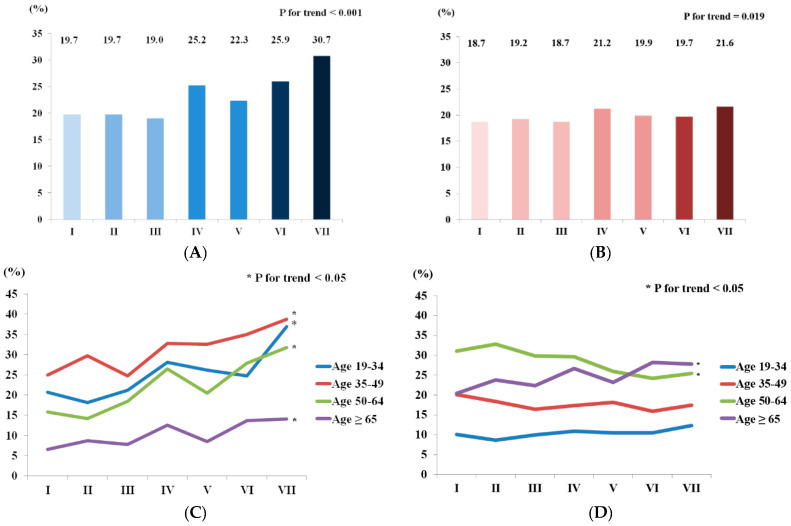
Trends in the prevalence of NAFLD in Korean men and women, KNHANES waves I-VII. (**A**) Prevalence of NAFLD in Korean men; (**B**) prevalence of NAFLD in Korean women; (**C**) prevalence of NAFLD in Korean men according to age group; (**D**) prevalence of NALFD in Korean women according to age group. NAFLD, non-alcoholic fatty liver disease; KNHANES, Korea National Health and Nutrition Examination Survey.

Figure 1 shows the trends in the prevalence of NAFLD in Korean men and women. The NAFLD prevalence among men has increased by approximately 11 percentage points in the past 19 years, reaching 30.7 ± 1.4% in wave VII. This increasing trend was observed in all age groups. The prevalence of NAFLD was higher in younger age groups. The prevalence in wave VII was 36.9 ± 3.6%, 38.8 ± 2.7%, 31.8 ± 2.7%, and 14.1 ± 1.5% in those aged 19–34, 35–49, 50–64, and ≥60 years, respectively. The NAFLD prevalence among women has increased by approximately three percentage points in the past 19 years. When the trend was analyzed according to age groups, the increasing trend was only observed in those aged ≥ 60 years.

Table 2 shows the trends in the prevalence of obesity, abdominal obesity, high fat intake, and low physical activity in Korean men and women. The prevalence of all associated factors of NAFLD has increased among men. The prevalence of obesity, abdominal obesity, high fat intake, and low physical activity in wave VII was 39.8 ± 1.5%, 33.2 ± 1.5%, 14.7 ± 1.3%, and 59.0 ± 1.5%, respectively. In the case of women, the prevalence of obesity, high fat intake, and low physical activity has increased; however, the trend in the prevalence of abdominal obesity was not significant.

Table 3 shows the ORs and 95% CIs for NAFLD and associated factors in each wave of KNHANES. In the multivariate model of men, the OR for NAFLD in wave VII was 1.93 (1.58–2.36) higher than that in wave I and showed increasing trend over time. Furthermore, the ORs for obesity, abdominal obesity, high fat intake, and low physical activity in wave VII were, respectively, 2.13 (1.77–2.58), 1.91 (1.56–2.35), 2.77 (2.00–3.85), and 4.97 (3.86–6.41) higher than those in the reference wave. They all showed increasing trends according to the wave of KNHANES. In the multivariate model of women, the ORs for NAFLD and abdominal obesity in wave VII were not significantly higher than those in wave I. The ORs for obesity, high fat intake and low physical activity in wave VII were 1.16 (1.02–1.31), 2.99 (2.44–3.67), and 5.02 (4.16–6.05) higher than those in the reference wave. Among these factors, high fat intake and low physical activity showed increasing trends.

### 3.3. Future Predictions for the Prevalence of NAFLD and Associated Factors in Korean Men

**Figure 2:**
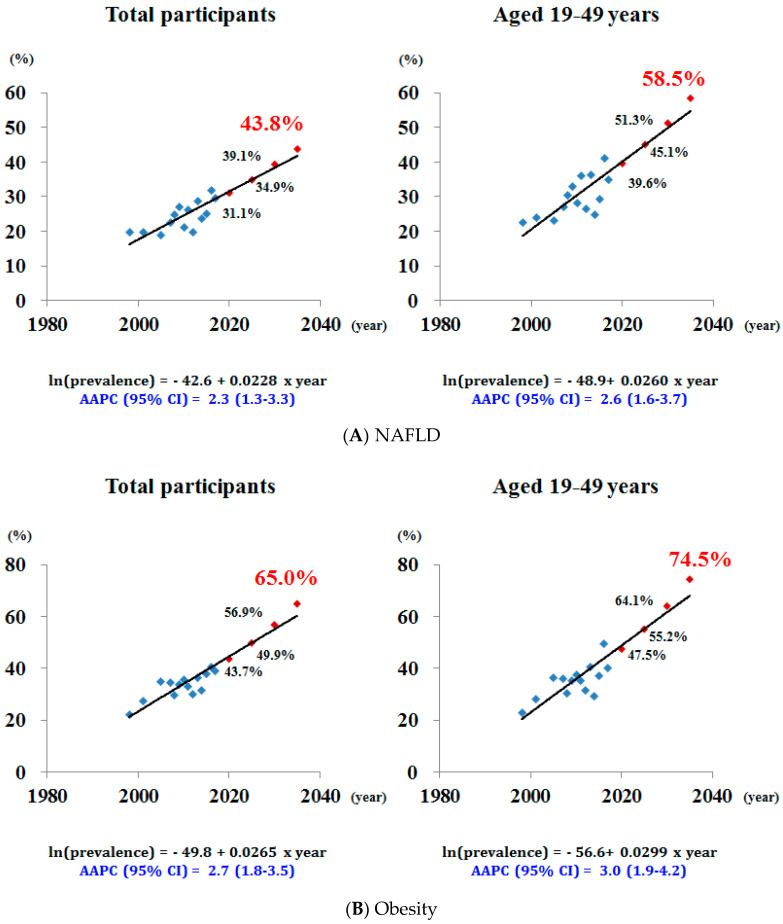

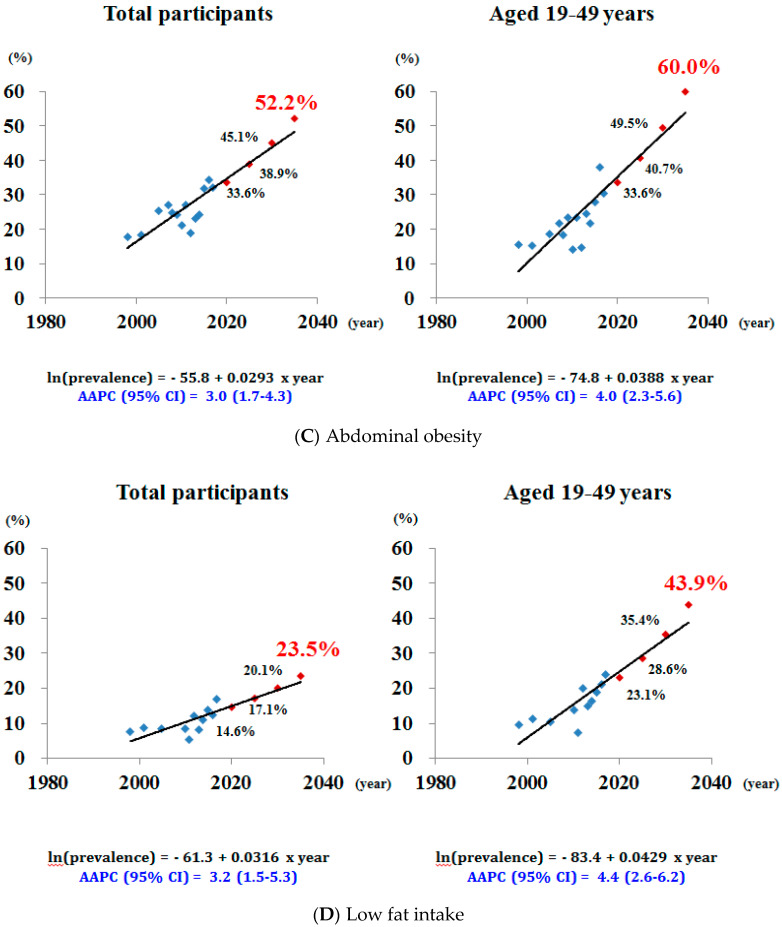
Future predictions using the joinpoint model for the prevalence of NAFLD and associated risk factors in the total participants and young Korean men. NAFLD, non-alcoholic fatty liver disease; AAPC, average annual percentage change.

Figure 2 shows the future predictions for the prevalence of NAFLD and associated factors in Korean men. The observed increase in NAFLD would result in a further increase in the prevalence of NAFLD such that approximately 40% of adult men will have NAFLD by 2030. The AAPC in the prevalence of NAFLD was 2.3% (1.33–3.3%) per year, and the predicted prevalence of NAFLD in 2035 was estimated to be nearly 44%. This increase was greater among the younger population of adult men aged ≤50 years. The AAPC in the prevalence of NAFLD was 2.6% (1.6–3.7%) per year among men aged 19–49 years, reaching 58.5% in 2035. The forecasted prevalence of obesity and abdominal obesity among Korean men was 65.0% and 52.2% in 2035. The increase was also greater among the younger population of men aged 19–49 years, reaching 74.5% and 60.0% in 2035. The predicted prevalence of high fat intake in Korean men was 23.5% in 2035 and in men aged 19–49 years was 45.2%.

## 4. Discussion

The prevalence of NAFLD in Korean men has rapidly increased with a linear trend from 1998 to 2017. This increase was accompanied by an increase in the prevalence of factors associated with NAFLD. Furthermore, the prevalence of obesity, abdominal obesity, and high fat intake will also increase with a linear trend in the future. The magnitude of increase was greater among the younger age group aged ≤50 years. The prevalence of NAFLD in Korean women has increased by approximately three percentage points in the past 19 years; however, this increase was not significant in the multivariate analysis.

According to our prediction model, the prevalence of NAFLD in Korean men will be approximately 40% in 2030 and 44% in 2035, exceeding the estimated future prevalence of NAFLD in other countries [25]. On the basis of a prediction model using data for the prevalence of obesity and type 2 diabetes mellitus, the estimated prevalence of NAFLD in 2030 was 28.4% in the US and 22.2% in China [25]. As the prevalence of NAFLD is lower in women [9], the forecasted prevalence in 2030 will be lower if women were included in our analysis. Nevertheless, it is obvious that the linear and rapid increase of NAFLD among Korean men will eventually result in decompensated cirrhosis, HCC, and liver-related deaths, thus creating a remarkably large public burden.

Obesity and abdominal obesity are major risk factors for NAFLD, and their prevalence has steadily increased in Korean men. This finding is consistent with previous studies in Korea and in other countries showing that the global prevalence of obesity has increased in the past years [26,27]. Obesity stimulates various inflammatory pathways in adipose tissue, disturbs insulin levels, and creates oxidative stress, which all play an essential role in the development of NAFLD [28,29]. Abdominal obesity is associated with an increase in visceral fat [28]. According to our forecast, the prevalence of obesity and abdominal obesity in Korean men will continue to increase. We estimated the prevalence of obesity to be 56.9% in 2030 and 65.0% in 2035. By 2030, an increase in the prevalence of obesity is expected in other countries including Australia, China, France, Germany, Italy, Japan, Spain, UK, and US [25,30,31]. The global increase in obesity will definitely influence the increase in future prevalence of NAFLD. In this study, the future prevalence of NAFLD among Korean men was estimated to be higher than that in Western countries, although the prevalence of obesity itself is lower in Korea [25]. This finding suggests a racial difference in the impact of obesity on NAFLD. Specifically, Asian populations are more susceptible to NAFLD development.

Although obesity is a strong risk factor of NAFLD, some patients with NAFLD have normal BMI. The condition referred to as lean NAFLD is more prevalent in Asians, and approximately 8–19% of Asian populations with normal BMI have NAFLD [32]. Although the histological disease severity in patients with lean NAFLD is still a matter of debate, these patients should not be overlooked as they share common altered cardiometabolic profiles with patients with obese NAFLD [32,33,34]. The rapid increase in the prevalence of NAFLD in both obese and lean patients will pose a public health threat.

Diet and physical activity are modifiable risk factors for NAFLD. Specifically, a Westernized diet with high fat and cholesterol levels induces NAFLD with fibrosis, and low physical activity, sedentary lifestyles, and prolonged sitting are associated with NAFLD [35,36,37]. Our findings demonstrate the consistent increase in the prevalence of high fat intake and low physical activity in Korean men. Lifestyle modifications including change in dietary habits and increased physical activity can improve the course of NAFLD. Diet rich in fibers, fish, and vegetables with low cholesterol and sugar, such as a Mediterranean diet, can improve NAFLD [38]. Furthermore, many clinical studies have shown the positive effect of aerobic and resistance training on NAFLD [38]. Appropriate lifestyle interventions should be undertaken. The prevalence of NAFLD-related factors, including obesity, abdominal obesity, high fat intake, and low physical activity, was lower in our study population than in the entire population of KNHANES. This implies that our predictions are not overestimated, and in fact, a higher prevalence of NAFLD and associated factors is expected in the real-world setting.

The prevalence of NAFLD was higher in the younger age group in this study. In waves I and VII, the prevalence of NAFLD among men aged 19–49 years was 22.6% and 37.9%, respectively, whereas, that among men aged ≥50 years was 12.5% and 23.6%, respectively. The predicted prevalence in 2035 among men aged 19–49 years was estimated to be 58.5%, which was greater than the prevalence among the entire adult male population. The past, current, and future NAFLD prevalence in Korean men remains higher in younger age groups, which contradicts the findings in other countries [39]. Previous studies in other countries consistently reported that the prevalence of NAFLD and related fibrosis increases with age [40,41,42,43]. The higher prevalence of NAFLD in younger age groups can cause further increase in the future prevalence of NAFLD as the younger generation shifts to the older generation over time. The constantly higher NAFLD prevalence in the younger age group can be explained by higher prevalence of factors associated with NAFLD among the younger population. According to the Korea Health Statistics, the prevalence of obesity in Korean adult men was constantly higher among men aged 19–49 years than among men aged ≥ 50 years from 2007 to 2017 [44]. Furthermore, a relatively rapid increase in the prevalence of abdominal obesity was observed in those aged 20, 30, and 40 years [26]. Moreover, the proportion of fat consumption relative to the total energy intake is constantly high among younger age groups from 2007 to 2017 [44]. A recent study reported poor control of lifestyle factors and cardiometabolic parameters in younger Korean adults [45]. Focus should be directed to modifiable lifestyle factors and accompanying metabolic disturbances, particularly among younger adults, to prevent a rapid increase in the prevalence of NAFLD in Korea.

In contrast to the rapidly increasing prevalence of NAFLD in Korean men, the prevalence of NAFLD in Korean women has not increased as much. The prevalence has increased approximately three percentage points over the past 19 years (18.7% in 1998 to 21.6% in 2017). This increase, in fact, was not significant after adjusting for potential confounders in the multivariate analysis. There are sex differences in the prevalence, risk factors, and clinical outcomes of NAFLD, which in particular mean that men have higher prevalence and worse outcomes [9,10,11]. This phenomenon could be explained by the role of estrogen. Estradiol reduces excess delivery of fatty acids to liver by preventing lipolysis and improving insulin sensitivity in adipose tissue and also protects from liver tumorigenesis [46,47,48].

Additionally, in terms of hepatic fibrosis, the risk of fibrosis defined by fibrosis-4 index [49] was 1.5%, 1.0%, 1.3%, and 1.6% in the wave IV, V, VI, and VII, respectively, among men in our study population. The prevalence of fibrosis did not change significantly during that period (p for trend 0.527). The study of hepatic fibrosis in a large population is needed in the future.

This study had several limitations. First, the criterion for NAFLD was not based on a gold-standard biopsy. However, it is difficult to apply a biopsy in a national survey. HSI is a suitable tool because it has high sensitivity and specificity for detecting NAFLD [20]. Many previous studies have used HSI as a measure of NAFLD [50,51,52]. Second, there is possibility of inaccuracy in the number of excluded participants as the KNHANES is a self-reported survey. We could neither detect autoimmune hepatitis status and nor distinguish acute and chronic hepatitis. Additionally, there might be inaccuracies in the number of excluded participants with a habit of drinking ≥30 g alcohol per occasion because recall bias in self-report may be present, especially the possibility of under-reporting of unhealthy lifestyles. Third, different tools were used to measure physical activity in this study. We were not able to measure physical activity for 1998 and 2001 as the KNHANES did not use a validated tool in that period. The IPAQ was used for the 2005–2013 data and the GPAQ was used for the 2014–2017 data, which may cause inconsistency in the definition of low physical activity. Fourth, it was not possible to differentiate controlled vs. uncontrolled, type 1 vs. type 2, and new onset vs. long standing in the KNHANES; however, this issue would not significantly affect our prediction model as the hepatic steatosis index needs diabetes as a categorical variable. Fifth, our analysis did not consider changes in treatment of the risk factors for NAFLD such as hypertension, diabetes mellitus, and dyslipidemia and changes in management of obesity which could affect future prevalence. Furthermore, our results should be interpreted with caution because population composition will be changed in the future. Moreover, the difference in the number of participants in each wave of KNHANES may have affected the results. Despite these limitations, our study is meaningful in that it reports the past, current, and future prevalence of NAFLD and associated risk factors.

## 5. Conclusions

In conclusion, the prevalence of NAFLD in Korean men has rapidly increased and will likely approach 40% in 2030 and 44% in 2035. The prevalence of associated risk factors for NAFLD in Korean men has also increased and will further increase in the future. Especially, in the younger age group, the prevalence of NAFLD and associated factors will rapidly increase, thus posing a considerable health threat. This study suggests that global public strategies as well as individualized approaches are urgently needed to prevent NAFLD and associated factors.

## Figures and Tables

**Table 1:** Basic characteristics of 40,948 study participants, Korea National Health and Nutrition Examination Survey (KNHANES) waves I–VII.

**Men (*n* = 10,870)**	**I (*n* = 2209)**	**II (*n* = 902)**	**III (*n* = 630)**	**IV (*n* = 1904)**	**V (*n* = 1953)**	**VI (*n* = 1755)**	**VII (*n* = 1517)**	***p* for Trend**
	Mean (SE)							
Height (cm)	169.2 (0.2)	169.3 (0.3)	168.0 (0.3)	168.5 (0.2)	169.1 (0.2)	169.3 (0.2)	169.9 (0.2)	0.007
Weight (kg)	65.7 (0.3)	66.9 (0.4)	67.0 (0.5)	67.5 (0.3)	68.1 (0.3)	69.0 (0.3)	71.0 (0.4)	<0.001
BMI (kg/m^2^)	22.9 (0.1)	23.3 (0.1)	23.7 (0.1)	23.7 (0.1)	23.8 (0.1)	24.0 (0.1)	24.5 (0.1)	<0.001
Waist circumference (cm)	81.9 (0.2)	83.0 (0.3)	83.3 (0.4)	83.7 (0.3)	83.4 (0.3)	84.2 (0.3)	86.1 (0.3)	<0.001
SBP (mmHg)	126.1 (0.5)	123.8 (0.7)	122.5 (0.8)	120.0 (0.5)	121.1 (0.4)	119.1 (0.4)	119.5 (0.4)	<0.001
DBP (mmHg)	79.9 (0.3)	78.7 (0.4)	79.5 (0.5)	78.0 (0.3)	77.6 (0.3)	75.7 (0.3)	76.7 (0.3)	<0.001
Fasting glucose (mg/dL)	100.4 (0.8)	96.8 (0.7)	95.5 (1.0)	98.4 (0.6)	99.2 (0.6)	101.0 (0.6)	102.2 (0.7)	<0.001
Total cholesterol (mg/dL)	185.3 (1.1)	188.0 (1.5)	182.9 (1.7)	184.3 (1.0)	187.7 (1.0)	185.6 (1.0)	190.4 (1.2)	0.010
AST (IU/L)	29.4 (0.5)	22.8 (0.3)	24.7 (0.4)	23.2 (0.3)	22.6 (0.3)	23.0 (0.3)	23.7 (0.3)	<0.001
ALT (IU/L)	33.7 (0.9)	23.7 (0.6)	26.7 (0.8)	25.8 (0.6)	24.2 (0.4)	25.3 (0.6)	27.6 (0.7)	<0.001
Energy intake (kcal/d)	2221.1 (32.1)	2149.5 (37.7)	2225.5 (45.6)	2073.8 (28.8)	2272.7 (29.0)	2271.6 (31.5)	2152.2 (34.7)	0.486
Carbohydrate intake (%)	66.3 (0.3)	65.1 (0.5)	65.2 (0.6)	69.9 (0.4)	68.1 (0.4)	65.9 (0.4)	65.5 (0.5)	0.661
Fat intake (%)	16.9 (0.3)	17.7 (0.4)	17.7 (0.5)	16.3 (0.3)	17.9 (0.3)	18.5 (0.3)	19.9 (0.3)	<0.001
Protein intake (%)	15.7 (0.2)	15.2 (0.2)	15.4 (0.2)	14.3 (0.1)	14.1 (0.1)	13.7 (0.1)	14.9 (0.2)	<0.001
	N (%)							
Low physical activity	-	-	158 (24.0)	635 (33.7)	843 (42.2)	870 (47.6)	919 (59.0)	<0.001
Drinking < 30 g alcohol/occasion	1620 (75.8)	592 (67.7)	328 (55.0)	894 (48.6)	1016 (52.1)	884 (51.8)	792 (54.5)	<0.001
Current smoking	1323 (60.4)	429 (49.1)	217 (34.0)	554 (30.6)	511 (29.1)	457 (28.3)	338 (23.0)	<0.001
Low income	479 (17.9)	208 (21.7)	184 (24.6)	526 (21.2)	508 (20.5)	415 (17.6)	355 (18.6)	0.303
Education < 12 years	1637 (68.7)	604 (62.4)	473 (72.5)	1449 (70.8)	1410 (68.9)	1207 (63.7)	913 (56.7)	<0.001
NAFLD	410 (19.7)	178 (19.7)	125 (19.0)	413 (25.2)	365 (22.3)	410 (25.9)	412 (30.7)	<0.001
Diabetes mellitus	265 (10.5)	89 (8.3)	72 (7.3)	262 (11.8)	304 (12.1)	287 (12.9)	260 (13.3)	<0.001
**Women (*n* = 30,078)**	**I (*n* = 4013)**	**II (*n* = 2784)**	**III (*n* = 2085)**	**IV (*n* = 5954)**	**V (*n* = 6221)**	**VI (*n* = 5093)**	**VII (*n* = 3928)**	***p* for Trend**
	Mean (SE)							
Height (cm)	156.4 (0.2)	156.2 (0.2)	156.3 (0.2)	156.0 (0.1)	156.4 (0.1)	157.1 (0.1)	156.8 (0.1)	<0.001
Weight (kg)	56.3 (0.1)	56.6 (0.2)	57.0 (0.3)	56.8 (0.1)	57.1 (0.2)	57.2 (0.2)	57.6 (0.2)	<0.001
BMI (kg/m^2^)	23.0 (0.1)	23.2 (0.1)	23.3 (0.1)	23.4 (0.1)	23.4 (0.1)	23.2 (0.1)	23.4 (0.1)	0.002
Waist circumference (cm)	77.6 (0.2)	78.1 (0.3)	78.0 (0.3)	78.8 (0.2)	78.3 (0.2)	77.9 (0.2)	78.9 (0.2)	0.009
SBP (mmHg)	122.5 (0.5)	119.8 (0.5)	117.1 (0.6)	116.4 (0.3)	118.6 (0.3)	116.0 (0.3)	117.9 (0.4)	<0.001
DBP (mmHg)	75.8 (0.3)	74.8 (0.3)	75.0 (0.4)	74.3 (0.2)	74.2 (0.2)	72.9 (0.2)	73.6 (0.2)	<0.001
Fasting glucose (mg/dL)	99.6 (0.7)	96.2 (0.5)	93.6 (0.7)	96.3 (0.3)	95.7 (0.3)	97.6 (0.4)	98.8 (0.4)	0.241
Total cholesterol (mg/dL)	186.4 (0.8)	187.4 (0.8)	184.8 (1.0)	189.5 (0.6)	190.5 (0.6)	190.1 (0.6)	195.2 (0.6)	<0.001
AST (IU/L)	24.4 (0.2)	20.4 (0.2)	21.8 (0.2)	20.5 (0.2)	20.1 (0.1)	20.3 (0.2)	20.8 (0.2)	<0.001
ALT (IU/L)	22.1 (0.3)	17.0 (0.3)	17.8 (0.3)	18.3 (0.2)	17.5 (0.2)	17.7 (0.2)	18.0 (0.2)	<0.001
Energy intake (kcal/day)	1751.0 (19.1)	1744.4 (20.0)	1773.0 (20.6)	1572.0 (10.8)	1682.9 (12.0)	1748.4 (12.4)	1618.4 (15.1)	<0.001
Carbohydrate intake (%)	69.4 (0.3)	68.5 (0.3)	66.6 (0.3)	71.6 (0.2)	70.4 (0.2)	67.0 (0.2)	67.8 (0.3)	<0.001
Fat intake (%)	16.1 (0.2)	16.0 (0.2)	17.5 (0.3)	15.6 (0.2)	16.7 (0.2)	18.4 (0.2)	19.6 (0.2)	<0.001
Protein intake (%)	15.4 (0.3)	14.4 (0.1)	15.1 (0.2)	14.0 (0.1)	14.0 (0.1)	13.6 (0.1)	14.3 (0.1)	<0.001
	N (%)							
Low physical activity	-	-	567 (27.1)	2378 (40.4)	3152 (51.0)	2938 (55.8)	2552 (65.1)	<0.001
Drinking < 30 g alcohol/occasion	2054 (54.5)	1499 (55.5)	1151 (58.3)	2764 (49.3)	3140 (53.3)	2573 (53.3)	2010 (53.9)	0.164
Current smoking	254 (5.9)	113 (3.9)	69 (3.5)	210 (3.8)	179 (3.4)	130 (2.7)	90 (2.5)	<0.001
Low income	924 (19.7)	668 (24.0)	544 (22.3)	1502 (20.1)	1392 (20.0)	1072 (17.3)	914 (19.8)	0.005
Education < 12 years	3361 (80.3)	2196 (76.4)	1644 (75.7)	4722 (75.6)	4665 (72.5)	3592 (66.2)	2540 (63.0)	<0.001
NAFLD	819 (18.7)	542 (19.2)	388 (18.7)	1322 (21.2)	1266 (19.9)	1088 (19.7)	903 (21.6)	0.019
Diabetes mellitus	378 (8.5)	241 (7.9)	161 (7.3)	630 (9.4)	641 (9.1)	580 (9.4)	532 (11.9)	<0.001

KNHANES, Korea National Health and Nutrition Examination Survey; BMI, body mass index; SBP, systolic blood pressure; DBP, diastolic blood pressure; AST, aspartate transaminase; ALT, alanine aminotrasnferase; NAFLD, non-alcoholic fatty liver disease.

**Table 2:** Comparison of basic characteristics between the study population and the total adults in KNHANES waves I–VII.

	I	II	III	IV	V	VI	VII	*p* for Trend
10,870 men in the present study								
BMI ≥ 25 kg/m^2^	494 (22.3)	260 (27.4)	224 (34.9)	570 (32.2)	617 (32.9)	613 (35.3)	573 (39.8)	<0.001
Waist circumference ≥ 90 cm	410 (17.8)	190 (18.3)	174 (25.4)	496 (25.0)	473 (22.2)	476 (26.4)	492 (33.2)	<0.001
Fat intake ≥ 30%	115 (7.6)	60 (8.8)	35 (8.4)	71 (6.1)	101 (8.8)	124 (11.0)	141 (14.7)	<0.001
Low physical activity	-	-	158 (24.0)	635 (33.7)	843 (42.2)	870 (47.6)	919 (59.0)	<0.001
Total adult men in KNHANES								
BMI ≥ 25 kg/m^2^	882 (25.0)	855 (31.1)	811 (34.7)	2546 (36.0)	2716 (35.9)	2753 (38.3)	2177 (41.5)	<0.001
Waist circumference ≥ 90 cm	713 (19.4)	634 (21.6)	599 (24.2)	1963 (25.4)	2048 (25.0)	2056 (27.1)	1750 (31.7)	<0.001
Fat intake ≥ 30%	194 (7.8)	200 (9.5)	171 (11.0)	347 (7.4)	535 (10.6)	565 (11.8)	549 (16.0)	<0.001
Low physical activity	-	-	489 (21.7)	2334 (31.7)	3115 (40.0)	3142 (45.7)	3042 (56.9)	<0.001
30,078 women in the present study								
BMI ≥ 25 kg/m^2^	1110 (25.4)	789 (27.4)	626 (28.7)	1814 (28.4)	1868 (29.0)	1481 (26.8)	1245 (30.0)	0.015
Waist circumference ≥ 85 cm	966 (21.6)	692 (24.2)	529 (24.3)	1771 (26.1)	1675 (25.0)	1254 (21.5)	1160 (26.7)	0.105
Fat intake ≥ 30%	224 (7.5)	174 (7.4)	143 (8.7)	255 (5.8)	405 (8.0)	456 (11.4)	493 (15.6)	<0.001
Low physical activity	-	-	567 (27.1)	2378 (40.4)	3152 (51.0)	2938 (55.8)	2552 (65.1)	<0.001
Total adult women in KNHANES								
BMI ≥ 25 kg/m^2^	1199 (25.4)	997 (27.1)	923 (28.0)	2938 (27.1)	3126 (28.3)	2869 (27.2)	2133 (28.9)	0.018
Waist circumference ≥ 85 cm	1046 (21.7)	854 (23.5)	764 (23.4)	2825 (24.6)	2771 (24.1)	2487 (22.8)	1969 (25.6)	0.052
Fat intake ≥ 30%	240 (7.4)	253 (8.9)	232 (9.4)	538 (7.3)	813 (9.9)	924 (12.6)	904 (16.8)	<0.001
Low physical activity	-	-	835 (27.1)	3987 (40.3)	5128 (49.2)	5140 (54.9)	4240 (61.9)	<0.001

Values are presented as unweighted numbers (weighted percentages). KNHANES, Korea National Health and Nutrition Examination Survey; BMI, body mass index.

**Table 3:** Odds ratios (ORs) and 95% confidence intervals (CIs) for NAFLD and associated factors in the study participants, KNHANES waves I–VII.

	I	II	III	IV	V	VI	VII	*p* for Trend
**Men**								
Univariate								
NAFLD	1.00	1.00 (0.801–0.26)	0.96 (0.751–0.23)	1.38 (1.151–0.66)	1.17 (0.961–0.42)	1.42 (1.191–0.71)	1.80 (1.502–0.17)	<0.001
Obesity	1.00	1.31 (1.071–0.61)	1.86 (1.472–0.36)	1.65 (1.391–0.97)	1.70 (1.432–0.03)	1.90 (1.602–0.25)	2.30 (1.932–0.74)	<0.001
Abdominal obesity	1.00	1.03 (0.821–0.30)	1.58 (1.232–0.02)	1.54 (1.281–0.85)	1.32 (1.091–0.58)	1.65 (1.381–0.98)	2.30 (1.902–0.78)	<0.001
High fat intake	1.00	1.16 (0.811–0.68)	1.12 (0.691–0.81)	0.79 (0.551–0.14)	1.16 (0.841–0.61)	1.50 (1.102–0.04)	2.09 (1.562–0.79)	<0.001
Low physical activity	-	-	1.00	1.61 (1.272–.05)	2.32 (1.822–0.94)	2.88 (2.273–0.65)	4.57 (3.585–0.84)	<0.001
Multivariate *								
NAFLD	1.00	0.97 (0.761–0.22)	0.94 (0.721–0.21)	1.48 (1.221–0.80)	1.22 (0.991–0.50)	1.53 (1.261–0.85)	1.93 (1.582–0.36)	<0.001
Obesity	1.00	1.25 (1.021–0.54)	1.67 (1.312–0.12)	1.55 (1.291–0.85)	1.56 (1.301–0.88)	1.74 (1.462–0.08)	2.13 (1.772–0.58)	<0.001
Abdominal obesity	1.00	0.93 (0.741–0.18)	1.31 (1.021–0.68)	1.28 (1.061–0.55)	1.07 (0.881–0.30)	1.38 (1.151–0.66)	1.91 (1.562–0.35)	<0.001
High fat intake	1.00	1.23 (0.841–0.80)	1.37 (0.832–0.26)	1.10 (0.751–0.61)	1.61 (1.142–0.29)	2.18 (1.583–0.01)	2.77 (2.003–0.85)	<0.001
Low physical activity	-	-	1.00	1.67 (1.302–0.15)	2.41 (1.883–0.09)	3.03 (2.373–0.88)	4.97 (3.866–0.41)	<0.001
**Women**								
Univariate								
NAFLD	1.00	1.04 (0.891–0.20)	1.01 (0.841–0.21)	1.17 (1.041–0.33)	1.09 (0.961–0.23)	1.07 (0.951–0.21)	1.20 (1.051–0.38)	0.019
Obesity	1.00	1.11 (0.971–0.26)	1.18 (1.011–0.38)	1.16 (1.041–0.30)	1.20 (1.071–0.34)	1.07 (0.961–0.20)	1.26 (1.111–0.42)	0.015
Abdominal obesity	1.00	1.16 (1.001–0.35)	1.16 (0.981–0.38)	1.28 (1.131–0.46)	1.21 (1.071–0.37)	1.00 (0.881–0.13)	1.33 (1.161–0.52)	0.105
High fat intake	1.00	1.00 (0.791–0.26)	1.18 (0.891–0.56)	0.77 (0.620–0.96)	1.08 (0.881–0.32)	1.60 (1.321–0.95)	2.30 (1.902–0.78)	<0.001
Low physical activity	-	-	1.00	1.82 (1.512–0.18)	2.79 (2.333–0.35)	3.40 (2.834–0.07)	5.02 (4.166–0.06)	<0.001
Multivariate *								
NAFLD	1.00	1.03 (0.881–0.19)	0.99 (0.821–0.19)	1.06 (0.931–0.21)	1.01 (0.891–0.15)	1.03 (0.911–0.17)	1.14 (0.991–0.32)	0.141
Obesity	1.00	1.08 (0.951–0.23)	1.13 (0.961–0.32)	1.05 (0.931–0.17)	1.10 (0.981–0.23)	1.01 (0.891–0.13)	1.16 (1.021–0.31)	0.328
Abdominal obesity	1.00	1.09 (0.941–0.26)	1.05 (0.891–0.25)	1.06 (0.931–0.21)	1.01 (0.881–0.15)	0.85 (0.740–0.97)	1.08 (0.941–0.25)	0.155
High fat intake	1.00	1.05 (0.831–0.33)	1.32 (1.001–0.75)	0.95 (0.761–0.18)	1.31 (1.061–0.61)	1.90 (1.552–0.34)	2.99 (2.443–0.67)	<0.001
Low physical activity	-	-	1.00	1.79 (1.492–0.15)	2.81 (2.343–0.37)	3.46 (2.884–0.15)	5.02 (4.166–0.05)	<0.001

* Multivariate model was adjusted for age, income, education, alcohol use, and smoking status. NAFLD, non-alcoholic fatty liver disease; KNHANES, Korea National Health and Nutrition Examination Survey.
